# Nursing Role in the Management of Major Ambulatory Surgery Units: A Scoping Review

**DOI:** 10.3390/healthcare14193289

**Published:** 2026-10-02

**Authors:** Lidia María Herrera-Aguiar, Jesús Manuel García-Acosta, Johan Gregorio Reyes-Quesada, Chaxiraxi Bacallado-Rodríguez, Silvia Elisa Razetto-Ramos, Elena Herrera-Aguiar, Francisco Javier Castro-Molina, Mª Mercedes Novo-Muñoz, Natalia Rodríguez-Novo

**Affiliations:** 1 Research Group on Care Throughout the Life Course—Health Management, Canary Islands Institute of Health Research (IISC), 38009 Santa Cruz de Tenerife, Spain; lherragu@ull.edu.es (L.M.H.-A.); jreyque@gobiernodecanarias.org (J.G.R.-Q.); cbacalla@ull.edu.es (C.B.-R.); srazetto@ull.edu.es (S.E.R.-R.); fcastrom@ull.edu.es (F.J.C.-M.); mernov@ull.edu.es (M.M.N.-M.); nrodrigu@ull.edu.es (N.R.-N.); 2 Nuestra Señora de Candelaria School of Nursing, University of La Laguna, 38010 Santa Cruz de Tenerife, Spain; 3 Canary Islands Health Service, 38071 Santa Cruz de Tenerife, Spain; eheraguy@gobiernodecanarias.org; 4 Faculty of Nursing, University of La Laguna, 38200 San Cristóbal de La Laguna, Spain

**Keywords:** ambulatory surgical procedures, nursing, health management, nursing role

## Abstract

**Background:** Major Ambulatory Surgery is an increasingly common care model, but evidence on nurses’ specific organizational roles remains limited. **Objectives:** To identify and map evidence on nursing roles in the organization, management, and optimization of MAS units. **Methods:** A scoping review was conducted according to Joanna Briggs Institute methodology and PRISMA-ScR guidelines. Embase, MEDLINE ALL (Ovid), CINAHL Complete, and Web of Science Core Collection were searched on 30–31 July 2025. Following an exploratory phase, the eligibility period was set from January 2000 to July 2025. Observational, qualitative, interventional, and review studies published in English or Spanish were included. **Results:** Of 922 records identified, 18 sources were included: 16 primary studies and two literature reviews, the latter analyzed separately as contextual evidence. The primary studies most frequently documented nursing roles in preoperative assessment, patient education, discharge preparation, postoperative follow-up, continuity of care, and care-process coordination. Six primary studies measured a service-level indicator (day-of-surgery cancellations, waiting times, or theatre utilization), all with uncontrolled before-after or descriptive designs; only one study addressed management, staffing, leadership, or organizational planning. Direct evidence on managerial, strategic, and organizational-planning roles was scarce. **Conclusions:** The central finding is the scarcity of evidence on nurses’ managerial, strategic, and organizational-planning roles, in contrast with the well-documented roles in preoperative assessment, patient education, postoperative follow-up, and care coordination. Because methodological quality was not appraised, the findings do not establish the effectiveness or comparative superiority of specific nursing models. Future research should define and evaluate nursing competencies and organizational models in MAS using robust comparative, qualitative, and mixed-methods designs.

## 1. Introduction

Major Ambulatory Surgery (MAS) has become firmly established over recent decades as an effective, safe, and patient-centered surgical care model that enables surgical procedures to be performed with same-day discharge and without the need for inpatient hospital admission [1].

Although its contemporary development is relatively recent, the origins of MAS can be traced to the early twentieth century, when James Nicoll laid the foundations for this model of care by performing ambulatory surgical procedures in pediatric patients at the Sick Children’s Hospital and Dispensary in Glasgow. In 1909, he reported favorable outcomes for 8988 surgical procedures performed without inpatient hospital admission [2].

The expansion of MAS has been driven by advances in minimally invasive surgery, improvements in anesthetic techniques, and the implementation of enhanced recovery protocols. It has also helped address the need to optimize healthcare resources, reduce surgical waiting lists, and respond to the steadily increasing demand for surgical procedures [3].

In this context, MAS has become a key strategy for improving healthcare system efficiency, reducing surgical waiting lists, and maintaining high standards of care [4]. In Spain, the progressive implementation of MAS since the 1990s has substantially transformed the organization of surgical services. Numerous studies have reported significant clinical and organizational benefits, including reduced hospital length of stay, lower rates of healthcare-associated infections, more efficient use of healthcare resources, and high levels of patient satisfaction [5,6]. Furthermore, ambulatory surgery rates have gradually increased across multiple surgical specialties. However, the range of procedures offered varies considerably among hospitals and across Spain’s autonomous communities, highlighting the influence of differences in organizational models, resource availability, and the internal management of MAS units [4,7].

The successful implementation and consolidation of MAS depend not only on technical advances but also on the efficient organization of care processes. This requires well-defined clinical pathways, appropriate patient selection, standardized protocols, effective coordination across different levels of care, and efficient management of operating room time and human resources within these units [8]. Therefore, MAS should not be regarded merely as a surgical modality but rather as a complex organizational model that requires careful planning, clinical leadership, and a culture of continuous quality improvement focused on patient safety and high-quality care [9].

Within the organizational framework of MAS, nursing plays a pivotal role in organizing and coordinating the care pathway, extending well beyond direct clinical care. Nurses are actively involved in surgical planning, the management of preoperative and postoperative care, patient and family education, and coordination of continuity of care after discharge [4].

From a management perspective, nurses play an integrative role among the professionals working within MAS, facilitating interdisciplinary communication and supporting the delivery of high-quality care. In addition, their involvement in organizing preanesthetic assessment clinics, implementing clinical protocols, and evaluating patient outcomes and satisfaction contributes significantly to the effective functioning of these units [10,11,12].

Despite this pivotal role, several authors have identified deficiencies in the specialized training of healthcare professionals working in MAS units. Furthermore, the absence of structured training pathways and clearly defined scopes of practice may contribute to variability in clinical practice and limit staff members’ professional development within these units. These issues are becoming increasingly important in light of the continued expansion of MAS, the growing number of patients treated, and the increasing complexity of surgical procedures performed in this setting [13,14].

The existing literature addresses the role of nursing in MAS from a variety of perspectives, but three gaps remain. First, nursing roles have been described mainly through single interventions at specific stages of the care pathway, such as patient satisfaction, postoperative care, or health education, without an integrated overview of the roles, competencies, and responsibilities exercised across the whole unit [8,9,10]. Second, the evidence is spread across heterogeneous designs, healthcare settings, and levels of intervention, which hinders the identification of the nursing roles and competencies relevant to the organization and operational efficiency of MAS units [5,8]. Third, it is unclear to what extent nurses’ organizational and managerial contributions, as opposed to their clinical activities, have been evaluated against service-level indicators. A preliminary search of the Open Science Framework (OSF), the Cochrane Library, JBI COnNECT+, and the Centre for Reviews and Dissemination (CRD) identified no scoping or systematic review addressing these questions.

A scoping review is the appropriate design for this purpose because the field is broad, conceptually heterogeneous, and covered by diverse study designs; it allows key concepts to be identified, the way in which the topic has been investigated to be examined, and knowledge gaps to be located [15,16].

Accordingly, the aim of this scoping review was to identify and map the available evidence on the role of nurses in the organization, management, and optimization of MAS units. The specific objectives were as follows:

1. To describe the roles, responsibilities, and competencies of nurses documented in MAS units;
2. To identify the organizational models and nurse-led services in which these roles are embedded;
3. To determine which organizational or service-level outcomes have been evaluated in relation to nursing roles, and with which study designs; and
4. To identify gaps in the literature regarding managerial, strategic, and organizational-planning roles.

## 2. Materials and Methods

### 2.1. Study Design

A scoping review was conducted in accordance with the Joanna Briggs Institute (JBI) methodology [15] and the Preferred Reporting Items for Systematic Reviews and Meta-Analyses Extension for Scoping Reviews (PRISMA-ScR) reporting guidelines [16].

The research question was formulated using the PCC (Population, Concept and Context) framework as follows: Population (nurses involved in MAS units); Concept (the roles, competencies, and responsibilities of nurses in the organization, management, and optimization of MAS); and Context (MAS units, including their organizational structure, same-day surgical care processes, and ambulatory healthcare settings). Accordingly, the review was guided by the following research question: What are the roles, competencies, and responsibilities of nurses in the organization, management, and optimization of MAS?

This scoping review was not prospectively registered. To support transparency and reproducibility, the search documentation was deposited in the Open Science Framework (OSF) repository (https://doi.org/10.17605/OSF.IO/PEH6A). The repository contains the records exported from each database, the database-specific search strategies (Supplementary Table S1), the PRISMA-ScR checklist, and a time-stamped search-chronology note, summarized in the project wiki. This note documents that the exploratory search restricted to the previous four years was performed on 28 February 2025, that the eligibility period was extended to January 2000–July 2025 on the same date, before any title and abstract screening had started, and that the final searches were performed on 30 and 31 July 2025. The repository was created on 17 April 2026, after the searches; the chronology is therefore documented from the review team’s records rather than as a prospectively registered amendment. The completed PRISMA-ScR checklist [16] is available as Supplementary File S1.

### 2.2. Identification of Relevant Studies and Search Strategy

A database-specific search strategy was developed in accordance with the Joanna Briggs Institute methodology for scoping reviews and the PRISMA-ScR reporting guidelines. The search strategy combined controlled vocabulary and free-text terms across three conceptual domains: major ambulatory surgery, management and organization, and nursing. Controlled vocabulary and field codes were adapted to the indexing system and syntax of each database, including Emtree and title/abstract fields in Embase, MeSH and title/abstract fields in MEDLINE ALL via Ovid, CINAHL Complete Headings and text-word fields in CINAHL Complete, and the Topic field in Web of Science Core Collection.

An initial exploratory search was conducted before the formal review process and was initially limited to publications from the previous four years. This preliminary search identified four potentially relevant studies. The publication-date range was subsequently expanded as a methodological amendment during the exploratory phase, before the formal screening of titles and abstracts, full-text assessment, or data extraction began. This decision was made to avoid restricting the evidence based on the limited yield of the initial exploratory search and to enable a more comprehensive mapping of the available literature.

No publication-date restriction was applied during the final database searches. The eligibility period from January 2000 to July 2025 had been established before formal screening and was subsequently applied during the screening process. This temporal delimitation was defined to capture the contemporary development of major ambulatory surgery and the evolution of nursing roles associated with this model of care. As contextual reference, major ambulatory surgery in Spain experienced a phase of maturity from approximately 1999 onwards, as described by Vila-Blanco [5].

The final searches were conducted in Embase via Elsevier, MEDLINE ALL via Ovid, CINAHL Complete via EBSCOhost and the Web of Science Core Collection, specifically the Science Citation Index Expanded (SCI-Expanded) on 30 and 31 July 2025. The final search therefore covered publications from 1 January 2000 through 31 July 2025. After completion of these searches, formal screening of all retrieved records was initiated in accordance with the predefined eligibility criteria. No title and abstract screening, full-text assessment, or data extraction was performed before the final publication period had been established. No additional sources were searched: grey literature (policy documents, national guidelines, conference proceedings, and trial registries) was not searched, reference lists of the included sources were not screened, and study authors were not contacted. This decision was taken when the search strategy was designed, to focus the map on empirical studies indexed in bibliographic databases; normative documents describe intended rather than observed nursing roles and were used only as context [4]. The single congress report included (Section 3.1) was retrieved through the database search.

The final searches retrieved 922 records: 297 from Embase, 41 from MEDLINE ALL via Ovid, 336 from CINAHL Complete, and 248 from Web of Science Core Collection. Table 1 summarizes the databases, platforms, search dates, limits, and retrieval counts. The complete database-specific search strategies, including controlled vocabulary, free-text terms, field codes, Boolean operators, limits, search dates, and retrieval counts, are presented in Supplementary Table S1.

Observational, qualitative, and interventional primary studies, as well as narrative and systematic reviews, published in English or Spanish, were eligible. The decision to retain literature reviews as contextual evidence, analyzed separately from the primary studies, was not specified as a priori; it was taken during title and abstract screening, when two reviews addressing the nursing role in MAS were identified (Section 3.1), and was applied consistently thereafter. Reviews were used to characterize how the nursing role in MAS had been synthesized to date and not as a source of primary empirical findings. To avoid double-counting, three rules were applied: review findings were charted in a separate block of the results tables (Section 3.1); reviews were excluded from all frequency and evidence-level counts and from citation strings referring to primary evidence; and, where a primary study included in this review had also been synthesized in one of the reviews, its findings were charted from the primary report only.

For the purposes of this review, an organizational or management nursing role was defined as any nursing responsibility or intervention that explicitly targets the coordination, standardization, planning, or optimization of MAS care processes at the level of the unit, pathway, or service, rather than the delivery of routine direct patient care alone. To be considered eligible, studies had to describe nursing activities that met at least one of the following criteria:

- Care-process coordination or pathway management, including preoperative assessment clinics, telephone-based triage, coordination of diagnostic tests and referrals, or management of patient flow through the MAS pathway;
- Development, implementation, or evaluation of standardized protocols, pathways, or organizational models, such as discharge criteria checklists, nurse-led preanesthetic assessment protocols, telemedicine-based preoperative assessment clinics, or nurse case-manager models;
- Resource or workflow optimization, including activities aimed at reducing same-day surgical cancellations, optimizing operating room time, improving staff allocation or nurse-to-patient ratios, or reducing waiting times across the surgical care pathway; or
- Quality improvement or organizational evaluation, including evaluation of nursing contributions to MAS efficiency, sustainability, or organizational outcomes (e.g., cancellation rates, length of stay, continuity of care), beyond the measurement of individual patient satisfaction or clinical outcomes alone.

Nursing activities that focused exclusively on the delivery of routine direct patient care (e.g., providing postoperative instructions without a standardized protocol, delivering usual postoperative follow-up without an organizational component, or assessing satisfaction without linking findings to care-process organization) were considered ineligible unless they were explicitly embedded within a broader organizational or quality-improvement initiative. This operational definition is consistent with the JBI approach to scoping reviews [15,16] which emphasizes the explicit specification of the concepts and boundaries of the evidence to be mapped. Based on this definition, all 18 included sources were verified to satisfy the eligibility criterion for an organizational or management nursing role. Studies that described only routine direct patient care without an explicit organizational, coordination, or quality-improvement component were excluded during full-text screening. Table 2 operationalizes each criterion with examples of activities that were included and of activities that were judged to be routine clinical care and excluded. The decisive feature in every criterion is that the activity targets a step of the MAS pathway at the level of the unit, pathway, or service (who is assessed, when, by whom, with which standardized instrument, and with what consequence for scheduling or discharge), rather than the delivery of care to an individual patient. Patient education and discharge interventions were therefore eligible only when delivered as a standardized protocol or instrument and evaluated as such. Studies that met a criterion solely through such an intervention, evaluated exclusively by patient-level outcomes (four studies, identified in Section 3.1.3), were labelled as being of borderline organizational relevance and were examined in a sensitivity check.

Studies that did not address the nursing role in the management or organization of MAS, as well as those focusing exclusively on other healthcare professionals or on clinical aspects without an organizational component, were excluded. Editorials, letters to the editor, and other publications lacking empirical data were also excluded, as were studies focusing on pediatric populations that did not address nursing management or organizational aspects.

All retrieved references were exported to the Rayyan (systematic review management platform) [17]. Duplicate records were identified through automated procedures and subsequently verified manually. After the removal of 119 duplicates, 803 records remained for title and abstract screening. Of these, 672 records were excluded, and 131 full-text reports were sought and assessed for eligibility. A total of 113 full-text reports were excluded because they did not address nursing management or organization (*n* = 83), did not address major ambulatory surgery (*n* = 10), or were ineligible publication types (*n* = 20). Eighteen studies were ultimately included in the review.

The final study selection process was conducted in two stages. First, titles and abstracts were screened according to the predefined eligibility criteria. Studies meeting the inclusion criteria, as well as those for which eligibility could not be determined from the title and abstract alone, proceeded to the full-text assessment stage. At both stages, two reviewers screened all records independently in Rayyan [17], with decisions blinded until the stage was completed. Inter-rater agreement was not quantified; conflicting decisions were unblinded and discussed until consensus was reached; agreement was achieved in all cases and recourse to a third reviewer was not required.

### 2.3. Data Extraction and Synthesis of Results

The findings identified through the literature review and data extraction were recorded in a purpose-designed, pilot-tested data extraction table to ensure its appropriateness and consistency. For the analysis, data were extracted on the main characteristics of the included studies, including authorship, year of publication, country of origin, study design, and study objective. In addition, specific information was collected on the nursing roles and responsibilities described within MAS units, together with the principal findings relevant to the research question. Data extraction was performed independently by two reviewers, and any discrepancies were resolved through consensus. No study authors were contacted to obtain or confirm data, and funding sources of the included studies were not charted.

The extracted data were synthesized using a descriptive and narrative approach. To distinguish the description of nursing practice from the evaluation of organizational effects, the evidence was organized across two analytical levels. The first level described the nursing roles, responsibilities, and activities reported in each source, including care-process coordination, preoperative assessment, patient education, discharge planning, follow-up, and case management. The second level identified whether the source evaluated organizational or service-level outcomes associated with these roles, such as surgical cancellations, waiting times, operating room efficiency or resource use, patient flow, pathway efficiency, service activity, or continuity of care. Patient-level outcomes (e.g., satisfaction, self-care confidence, health status, pain or anxiety, quality of recovery, adverse events) and audits of documentation quality were recorded separately and were not classified as organizational or service-level outcomes. The presence of a documented nursing role was therefore not interpreted as evidence of an organizational effect unless the study explicitly evaluated such an outcome. The thematic mapping was performed by the review team using the data recorded in the extraction table. The initial framework was informed by the review question and the PCC components and was refined inductively through comparison of the nursing roles, responsibilities, and competencies reported across the included sources. Disagreements regarding category allocation were discussed and resolved by consensus. Six non-mutually exclusive categories were identified: preoperative assessment and preparation; patient education and discharge planning; continuity of care and postoperative follow-up; care-process and patient-flow coordination; advanced nursing practice and nurse-led services; and management, staffing, leadership, and organizational planning.

A formal methodological quality assessment of the included studies was not undertaken, in accordance with the exploratory nature of this scoping review and the methodological recommendations of the JBI [15]. Accordingly, the findings were synthesized in a manner consistent with the purpose of scoping reviews, which is to map the available evidence and identify key concepts and knowledge gaps within the existing literature.

Of the 18 included sources, two were literature reviews [18,19], and the remaining 16 were primary empirical studies. To avoid double counting, the reviews were not used as a source of primary data. Instead, they were analyzed separately to identify how prior syntheses have described the nursing role in MAS and to highlight conceptual or organizational themes that had not been explicitly addressed in the primary studies. Findings from the reviews are therefore presented as contextual evidence rather than as additional empirical observations.

Because the role categories are non-mutually exclusive and the number of primary studies is small, category frequencies are reported as descriptive counts (*n* of 16) without percentages; they describe the distribution of the mapped literature and carry no inferential meaning. A sensitivity check was performed in which the four studies of borderline organizational relevance (Table 2, footnote) were excluded, and the category frequencies were recalculated for the remaining 12 primary studies. The overlap among categories is summarized in a co-occurrence matrix (Supplementary Table S2).

## 3. Results

**Figure 1:**
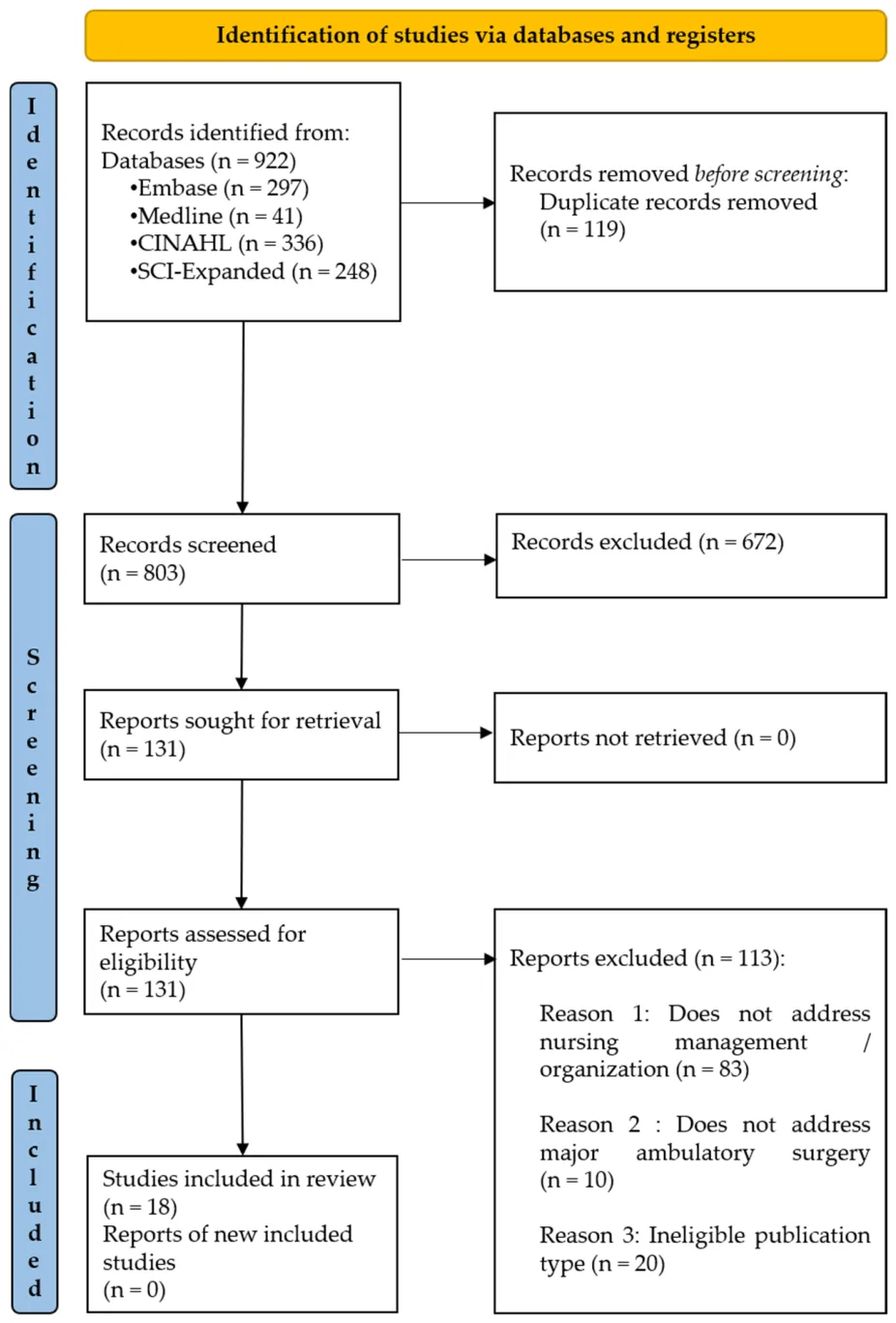
Flow diagram of the study identification, screening, eligibility, and inclusion process. Adapted from the Preferred Reporting Items for Systematic Reviews and Meta-Analyses Extension for Scoping Reviews (PRISMA-ScR).

The literature search identified a total of 922 records across the selected electronic databases. Following the study selection process, 18 sources met the eligibility criteria and were included in this scoping review. Figure 1 presents the PRISMA-ScR flow diagram [16], illustrating the complete study selection process.

### 3.1. Characteristics of the Included Studies

The 18 included sources comprised 16 primary empirical studies and two literature reviews. The primary studies showed considerable heterogeneity in terms of study design, geographical setting, and thematic focus, including descriptive, observational, cohort, retrospective, quasi-experimental, and qualitative designs. The two reviews were retained to characterize how the nursing role in MAS has been synthesized in prior literature and were analyzed separately from the primary evidence base [18,19].

Regarding geographical context, the included studies were conducted in a range of countries and healthcare systems, including the United States [20,21,22,23,24,25], China [26,27,28], Belgium [18], Qatar [29], Spain [30], Singapore [31], Canada [32], Sweden [33], the United Kingdom [19,34] and Italy [35].

This geographical diversity provides a broad perspective on the organization and operation of MAS units and the role of nursing within these settings.

Overall, the 16 primary studies examined various aspects of the nursing role in MAS, including patient education [20,21,26,27,29,30,33,34], discharge preparation [22,25,27,32], coordination of the care pathway [20,24,27,28,35], implementation of specific nursing interventions [35], and measurement of organizational or service-level indicators [21,23,27,30,31,34]. The two literature reviews [18,19] addressed nurse staffing models in ambulatory surgery units and the nurse’s role in day surgery, respectively; their contribution is described separately in Section 3.1.1 and Section 3.1.3.

Table 3 summarizes the characteristics of the included sources, sorted from most recent to least recent. Reviews are identified as such in the ‘Type of Study’ column, were not used as a source of primary empirical findings and are not included in the frequency counts reported below.

#### 3.1.1. Documented Nursing Roles and Responsibilities

The 16 primary studies described a broad range of nursing roles and responsibilities within MAS. The most frequently reported activities were preoperative assessment and preparation, patient education, discharge planning, postoperative follow-up, care-process coordination, telephone-based assessment, and case management [20,21,22,23,24,25,26,27,28,29,30,31,32,33,34,35]. Several primary studies described nursing involvement in the coordination of diagnostic tests and referrals, the management of patient flow, the application of discharge criteria, and the implementation of standardized perioperative pathways [20,21,25,27,28,31,34,35]. Considered separately as contextual evidence, the two literature reviews similarly identified patient preparation, education, discharge coordination, staffing, and patient-flow management as relevant nursing functions [18,19]. These findings describe the scope and content of nursing practice but do not, by themselves, demonstrate an organizational effect.

#### 3.1.2. Organizational and Service-Level Outcomes

Six of the 16 primary studies measured at least one organizational or service-level indicator [21,23,27,30,31,34] (Table 4): day-of-surgery cancellations [21,23,30,31], waiting times along the surgical pathway [27], and theatre utilization and non-attendance [34]. Four of these studies used uncontrolled before-after designs [21,23,27,31] and two were descriptive without a comparison group [30,34]; none included a concurrent control group. Six primary studies evaluated patient-level outcomes only, such as patient satisfaction, self-care confidence, health status, anxiety and pain, or quality of recovery [22,26,28,29,32,35]; this group includes the only study with a concurrent comparison of two wards, in which pathway efficiency was described but not measured [35]. Four studies provided role description without outcome evaluation [20,24,25,33]. Patient-level outcomes were reported separately from organizational indicators and were not interpreted as evidence of service-level efficiency. The staffing-model review [18] discussed nurse staffing and patient-flow considerations but did not evaluate outcomes and is not counted here. Overall, nursing roles are well documented, whereas direct measurement of organizational effects is limited to a small number of uncontrolled or descriptive studies.

#### 3.1.3. Thematic Mapping of Nursing Roles

The thematic mapping identified six non-mutually exclusive categories of nursing roles in MAS: preoperative assessment and preparation; patient education and discharge planning; continuity of care and postoperative follow-up; care-process and patient-flow coordination; advanced nursing practice and nurse-led services; and management, staffing, leadership, and organizational planning.

The most salient result of the thematic mapping is the asymmetry between clinical and managerial roles: only one of the 16 primary studies explicitly addressed management, staffing, leadership, or organizational planning [28], and this category was otherwise represented only by the staffing-model review [18]. This finding should be read with the caveat that the search did not include terms specifically targeting nurse managers or leadership roles (Section 4.3).

Table 5 cross-references each included source with the six role categories and with the evidence-level classification of Table 3. Frequencies are descriptive counts of the 16 primary studies. Patient education and discharge planning (13 of 16) and preoperative assessment and preparation (10 of 16) were the most frequently represented categories, followed by care-process and patient-flow coordination (9 of 16), continuity of care and postoperative follow-up (6 of 16), advanced nursing practice and nurse-led services (6 of 16), and management, staffing, leadership, and organizational planning (1 of 16). Categories overlap substantially: three studies were assigned to one category, three to two, seven to three, and three to four or more (median, three); the largest overlaps were between preoperative assessment and patient education (8 studies) and between preoperative assessment and care-process coordination (7 studies) (Supplementary Table S2). The two literature reviews are displayed separately in Table 5 and are not included in the counts.

In the sensitivity check excluding the four studies of borderline organizational relevance [22,24,26,32], the ranking of categories was essentially unchanged among the remaining 12 primary studies: preoperative assessment and preparation (10 of 12), patient education and discharge planning (9 of 12), care-process and patient-flow coordination (8 of 12), advanced nursing practice and nurse-led services (6 of 12), continuity of care and postoperative follow-up (5 of 12), and management, staffing, leadership, and organizational planning (1 of 12). The category most affected was patient education and discharge planning, as expected, whereas the management category remained represented by a single study.

## 4. Discussion

The 16 primary studies included in this review reported a predominantly clinical profile of nursing activities in MAS, particularly in patient education, discharge preparation, and care coordination. These findings describe the roles documented in the literature but do not establish their effectiveness or independent organizational impact. Six primary studies measured a service-level indicator (day-of-surgery cancellations, waiting times, or theatre utilization) [21,23,27,30,31,34]; four used uncontrolled before-after designs and two were descriptive, and none included a concurrent control group (Table 4). Because methodological quality was not formally appraised, these findings are presented as study-level reports and are not interpreted as evidence of comparative effectiveness or causal impact at the review level.

Four uncontrolled before-after studies reported reductions in day-of-surgery cancellations [21,23,31] or in waiting times [27] after the introduction of nurse-led preoperative assessment or pathway interventions, and one service-development report described improved theatre utilization without quantitative data [34]. These are study-level reports from designs that are vulnerable to secular trends, regression to the mean, and confounding by concurrent changes; because methodological quality was not appraised and outcome definitions were heterogeneous, they are not interpreted here as evidence of effectiveness. The present review maps which indicators have been measured and with which designs but cannot determine whether specific nursing models are more effective than alternatives or whether the reported changes were attributable to nursing interventions [21,23,27,30,31,34].

Furthermore, most studies focused on clinical outcomes and patient satisfaction, whereas few examined the relationship between nursing practice and organizational or healthcare system efficiency indicators [20,22,24,26,28,29,32].

In this regard, key components of MAS, such as patient flow optimization, reduction in surgical cancellations, surgical waiting list management, and the sustainability of the care model, remain underexplored from a nursing perspective, although nurses are routinely assigned responsibilities in these processes [21,23,27,28,31,34].

Overall, the mapped evidence is substantially stronger for nurses’ clinical and care-coordination functions (Table 5) than for their strategic, managerial, or leadership responsibilities. The limited direct evidence concerning resource allocation, waiting-list management, organizational planning, and service-level leadership represents a central finding of this review rather than evidence that these roles are already well established or adequately evaluated.

### 4.1. Identified Gaps

There is a lack of studies that systematically and consistently define nursing roles involved in the organization and management of MAS. Although many studies describe individual nursing interventions, few adopt a comprehensive perspective on nurses’ roles within the organizational and structural framework of the MAS model [24,26,27].

Furthermore, there is a lack of specific evidence regarding nurses’ roles in management, leadership, and process coordination. Although some primary studies describe the experiences of nurse coordinators or report indirect organizational contributions [29,33], and one review addressed nurse staffing models [18], the literature reveals a paucity of research examining nurse-led decision-making, strategic planning, resource management, and interprofessional coordination within MAS units.

In addition, limited evidence was identified on the direct impact of nurses’ roles on efficiency indicators and organizational outcomes, such as reductions in scheduled surgical cancellations, optimization of surgical waiting lists, length of stay, continuity of care, and sustainability of the MAS model [21,23,30,31,34].

Most studies focused on the clinical outcomes of procedures performed in MAS or on patient satisfaction with different aspects of the MAS care pathway, without explicitly linking these outcomes to organizational structures or nursing management models, which were the primary focus of this scoping review [19,22,24,29,32].

### 4.2. Implications for Practice and Research

The included studies describe nurses’ involvement in patient education, continuity of care, and quality-improvement activities [24,29]. Given the gaps identified, the development and evaluation of advanced nursing roles, such as a MAS nurse coordinator, is a hypothesis to be tested in future studies rather than a recommendation supported by the mapped evidence.

Furthermore, there is a clear need to develop organizational models and standardized protocols that explicitly integrate nursing roles into the planning, implementation, and evaluation of MAS processes. Defining specific competencies and developing training programs tailored to this care setting could promote more consistent, evidence-based practice [18].

Regarding research, this review highlights the need for studies that specifically examine the impact of nurses’ roles on organizational and management indicators beyond traditional clinical outcomes. Further research is needed to evaluate the contribution of nursing to healthcare system efficiency, resource optimization, reduction in adverse events, and sustainability of the MAS model [18,19]. Beyond organizational outcomes, future research should also address the organizational conditions under which nurses contribute to MAS processes. Workload, staffing and resource adequacy, and managerial leadership and support are measurable organizational factors that may influence nurses’ capacity to engage in care coordination, pathway management, and service organization. Evidence from other nursing settings shows that these factors can be operationalized with validated instruments and examined as determinants of care delivery; in a multicenter study of home care nurses, higher workload was associated with more missed nursing care, whereas staffing and resource adequacy and leadership quality were associated with less [36]. Although this evidence derives from a different care context, applying a comparable framework in MAS would allow future studies to examine not only efficiency indicators but also how workload, staffing adequacy, and managerial leadership shape nurses’ contribution to care coordination and, ultimately, the quality and safety of ambulatory surgical care.

Regarding study designs, randomization of nurse-led organizational models at the patient level is rarely feasible in MAS units. Priority should therefore be given to controlled before-after and interrupted time-series designs with enough pre- and post-implementation periods to account for secular trends, to stepped-wedge implementation when a model is rolled out across units of the same organization, to cluster-randomized designs where several comparable units are available, and to embedded qualitative or realist evaluation to explain how and under which conditions a model works. Regarding outcomes, a common set of service-level indicators that are routinely recorded in MAS units and were measured in the included studies should be adopted: day-of-surgery cancellation rate and its causes, non-attendance rate, waiting time from indication to surgery, operating room utilization and turnover time, unplanned admission and 30-day readmission rates, unit length of stay, and cost per case. These should be complemented by nurse-sensitive organizational measures (staffing adequacy, workload, missed nursing care) and by patient-reported experience measures.

Similarly, future qualitative studies could provide a deeper understanding of nurses’ experiences, perspectives, and recommendations for improvement, thereby informing the development of theoretical models and conceptual frameworks to guide clinical practice and organizational management in this field [18,28].

The transferability of the mapped roles to the Spanish MAS context, which motivated this review, is only partial. Nine of the 16 primary studies were conducted in the United States [20,21,22,23,24,25] or China [26,27,28], and only one in Spain [30]. The processes described (structured nurse-led preoperative assessment by telephone or clinic, standardized discharge criteria and information, telephone follow-up, coordination of tests and referrals) are transferable in principle, because they target steps of the MAS pathway that are common to the Spanish model defined in the national standards [4] and already exist, with local variation, in Spanish units [8,14]. The professional roles through which they are delivered are less transferable: several studies rely on advanced practice or nurse practitioner roles [20,23,31] whose scope of practice and regulatory recognition are still developing in Spain, the staffing models discussed in the contextual review [18] presuppose organizational structures that differ from those of Spanish public hospitals, and the Chinese studies describe high-volume day-surgery wards within tertiary hospitals rather than the integrated or autonomous units that predominate in Spain [8]. The mapped evidence therefore identifies transferable processes and indicators, but not directly transferable role models, and the evaluation of nurse-led organizational roles within the Spanish regulatory framework is itself one of the gaps identified.

### 4.3. Strengths and Limitations of the Review

One of the main strengths of this review is the use of a systematic methodology based on JBI guidance and reporting in accordance with the PRISMA-ScR guidelines, which enabled a comprehensive and structured mapping of the available evidence on the role of nursing in MAS.

The inclusion of studies employing different methodological designs and conducted across a range of international settings provided a comprehensive overview of the phenomenon under investigation. However, this review also has several limitations. First, the methodological heterogeneity of the included studies in terms of study design, objectives, and variables analyzed limits the direct comparison of findings and the formulation of generalizable conclusions. Furthermore, many of the included studies did not primarily aim to examine nursing roles but rather addressed them indirectly, which may have limited the depth of the analysis.

A formal methodological quality assessment of the included studies was not undertaken, in accordance with the exploratory purpose of this scoping review and the methodological recommendations of the JBI. Consequently, the review cannot determine the credibility, comparative effectiveness, or strength of the evidence supporting specific nursing models. Findings concerning reductions in cancellations, improvements in efficiency, patient satisfaction, or other outcomes are therefore presented as reported by the individual studies and should not be interpreted as causal or review-level conclusions.

Several further limitations concern the search and selection process. The search was limited to four bibliographic databases and to studies published in English or Spanish; grey literature, reference lists, and study authors were not consulted, which may have led to the omission of unpublished evaluations and of managerial descriptions circulating in professional and policy documents. Although the management block of the search combined general terms (management, organization, workflow, efficiency, implementation) with exploded nursing headings that encompass nurse administrators, it did not include terms specifically targeting nurse managers, nurse coordinators, leadership, or staffing; the scarcity of evidence in the management category should be read with this caveat. Inter-rater agreement during screening was not quantified, which limits the assessment of the reproducibility of the selection. The decision to retain literature reviews as contextual evidence was taken during screening rather than a priori, although it was applied consistently and reviews were excluded from all counts. Finally, the primary studies are geographically concentrated in the United States and China, with a single Spanish study, which limits the transferability of the mapped role models to other healthcare systems.

Despite these limitations, the findings provide a comprehensive overview of the role of nursing in MAS and contribute to identifying priority areas for the future development of research and clinical practice in this field.

## 5. Conclusions

The central finding of this scoping review is the asymmetry between the abundant description of nurses’ clinical and care-coordination roles in MAS and the scarcity of evidence on their managerial, strategic, and organizational-planning roles. Only one of the 16 primary studies explicitly addressed management, staffing, leadership, or organizational planning, and only six measured a service-level indicator, all with uncontrolled before-after or descriptive designs. Consequently, the available literature does not establish the organizational effectiveness or the comparative superiority of any nursing management model in MAS.

Within this limitation, the included studies consistently describe nursing involvement in preoperative assessment and preparation, patient education, discharge planning, postoperative follow-up, continuity of care, and care-process coordination, frequently delivered through nurse-led telephone or clinic-based services.

The evidence is heterogeneous, geographically concentrated, and predominantly quantitative and descriptive, with few qualitative studies exploring nurses’ professional experiences, and there is limited standardization of the competencies, professional profiles, and training models specific to MAS. The absence of theoretical frameworks integrating clinical and organizational responsibilities and the limited evaluation of efficiency and sustainability indicators identify important gaps in role definition, competencies, and validated training programs.

Future research should prioritize (i) the explicit definition of nursing competencies for MAS management and leadership; (ii) the evaluation of nurse-led organizational models with controlled before-after, interrupted time-series, or stepped-wedge designs; (iii) a common set of service-level indicators (day-of-surgery cancellations, non-attendance, waiting times, operating room utilization, unplanned admissions, and cost per case) complemented by nurse-sensitive organizational measures such as staffing adequacy, workload, and missed nursing care; and (iv) qualitative and mixed-methods studies of nurses’ experiences in coordinating and managing MAS units, including within the Spanish healthcare system.

## Figures and Tables

**Table 1:** Summary of the databases and records cited in the main manuscript.

Database	Platform	Search Date	Language Limits	Publication-Date Limits	Records Retrieved
Embase	Elsevier	31 July 2025	English or Spanish	None applied during search; January 2000–July 2025 applied during screening	297
MEDLINE All	Ovid	30 July 2025	English or Spanish	None applied during search; January 2000–July 2025 applied during screening	41
CINAHL Complete	EBSCOhost	31 July 2025	English or Spanish	None applied during search; January 2000–July 2025 applied during screening	336
Web of Science Core Collection SCI-Expanded	Web of Science	31 July 2025	English or Spanish	None applied during search; January 2000–July 2025 applied during screening	248
Total					922

**Table 2:** Operational definition of an organizational or management nursing role: criteria with examples of included and excluded activities.

Criterion	Defining Feature	Included Activities (Examples from Included Studies)	Excluded Activities (Routine Clinical Care)
1. Care-process coordination or pathway management	The nurse organizes a step of the pathway for a defined population (triage, scheduling, referral, patient flow).	Nurse-led preanesthetic assessment clinic with structured triage and referral; telephone preoperative assessment coordinating tests and referrals; nurse case manager coordinating the whole pathway; management of the patient pathway in a nurse-led ward model.	Accompanying or preparing an individual patient on the day of surgery; routine perioperative nursing care without responsibility for pathway steps.
2. Development, implementation, or evaluation of standardized protocols, pathways, or organizational models	A protocol, instrument, or model applied to all eligible patients and evaluated as such.	Discharge-criteria checklist; standardized or diagnosis-specific discharge information protocols; postoperative feedback-oriented education programme within an ambulatory pathway; evidence-based perioperative pathway; telemedicine preanesthetic clinic.	Individualized discharge teaching without a standardized protocol; procedure-specific clinical guidance without a nursing organizational component.
3. Resource or workflow optimization	The stated aim is a service indicator (cancellations, theatre time, staffing, waiting time).	Preoperative telephone calls targeting causes of day-of-surgery cancellation; analysis of nursing-related causes of surgical cancellation; advanced practice nurse-led telephone assessment to reduce cancellations.	Surgeon- or anesthetist-led scheduling studies without a nursing role; studies of analgesic or anesthetic techniques delivered by nurses.
4. Quality improvement or organizational evaluation	Nursing contribution assessed at unit or service level (audit, survey of routines, role description spanning the pathway).	Audit of a nurse-led preanesthetic service; national survey of nursing routines to identify improvement targets; qualitative study of nurses’ perspectives on the pain-management and education process; description of an advanced practice nurse role spanning the pathway.	Measurement of patient satisfaction or anxiety after usual care, not linked to a process change; evaluation of a clinical technique.

Studies that met a criterion solely through a standardized education or discharge intervention evaluated by patient-level outcomes were considered of borderline organizational relevance and were excluded in the sensitivity check (four studies, identified in Section 3.1.3). Examples are drawn from the included studies; the corresponding sources are identified in Section 3.1.

**Table 3:** Characteristics of the Included Studies.

First Author	Year	Country	Type of Study	Goal	Description of Nursing Roles and Responsibilities	Evidence Level	Key Findings
Wolgemuth E et al. [23]	2025	USA	Prospective quality-improvement project with pre- and post-implementation assessment	To implement a telemedicine-based preanesthetic assessment clinic to reduce same-day surgical cancellations.	Preoperative assessment conducted via teleconsultation using a standardized protocol, including a comprehensive clinical evaluation of the patient.	Role description and organizational/service-level outcome evaluation	Uncontrolled before-after assessment: day-of-surgery cancellations fell from 3.38% to 1.72% after implementation of the telehealth preanesthetic clinic.
Yao X et al. [26]	2025	China	Cohort study	To evaluate the effect of patient education on outcomes in patients undergoing ambulatory robotic adrenalectomy.	Postoperative patient education, follow-up, and support to promote adherence to the prescribed treatment plan.	Role description and patient-level outcome evaluation	The nursing-led educational intervention was associated with improved overall health status.
Van Caelenberg E et al. [18]	2023	Belgium	Literature review *	To identify appropriate nurse staffing models for MAS units.	Provision of postoperative care, patient education, discharge coordination, and management of patient flow.	Contextual review evidence	Limited evidence is available on optimal nurse staffing models. Further research is needed to establish appropriate staffing models and nurse-to-patient ratios.
Wu M et al. [27]	2023	China	Quasi-experimental study	To implement an evidence-based perioperative care pathway in MAS.	Preoperative screening, patient education, coordination of the surgical pathway, discharge assessment, and postoperative telephone follow-up.	Role description and organizational/service-level outcome evaluation	Uncontrolled before-after study: the authors reported shorter waiting times for admission and surgery and improved compliance with perioperative audit indicators.
Nallani R et al. [24]	2021	USA	Qualitative study	To explore perioperative nurses’ perspectives on pain management and patient education in MAS.	Assessment and management of pain, patient education, and coordination of perioperative care.	Role description	Deficiencies were identified in interdisciplinary communication and patient education.
Arun N et al. [29]	2021	Qatar	Observational study	To evaluate the feasibility and quality of a nurse-led preanesthetic assessment clinic in MAS.	Preanesthetic assessment, clinical interview, physical examination, risk stratification, and patient education.	Role description and patient-level outcome evaluation	An audit of 100 records showed 95.3% compliance with assessment standards; patient and nurse satisfaction were high, and no adverse events were reported. No cancellation, waiting-time, or resource-use outcomes were measured.
Higueras San Román BM [30]	2020	Spain	Descriptive study	To evaluate the effectiveness of nurse-led preoperative assessment by analyzing surgical cancellations in MAS.	Preoperative assessment, patient education, and perioperative preparation.	Role description and organizational/service-level outcome evaluation	Descriptive study (2018–2019): of 4013 procedures scheduled after nurse-led preoperative assessment, 103 (2.57%) were cancelled; medical causes (54.36%) and patient non-attendance (19.42%) predominated, and inadequate preoperative preparation accounted for 11.65% of cancellations, which the author linked to the nursing role in preoperative assessment.
Xu Y [28]	2018	China	Descriptive study	To explore a nurse-led management model within an MAS unit.	Management of the patient pathway, perioperative assessment, patient education, and postoperative follow-up.	Role description and patient-level outcome evaluation	Descriptive congress report of a nurse-led management model; the author reported a 6.8% improvement in patient satisfaction and a threefold increase in surgical activity (2013–2017), without formal evaluation of process outcomes.
Teh AHM et al. [31]	2016	Singapore	Quasi-experimental study	To evaluate an advanced practice nurse-led preoperative telephone assessment.	Telephone assessment, risk identification, and referral.	Role description and organizational/service-level outcome evaluation	Pre-post comparison without concurrent control (200 records before, 200 patients after): day-of-surgery cancellations decreased from 9 (4.5%) to none (*p* = 0.01) after implementation of the nurse-led telephone assessment; 10 patients required additional investigations and 7 were redirected to face-to-face assessment.
Lynch KM, LeFort SM [32]	2016	Canada	Descriptive study	To evaluate the effect of standardized, nurse-delivered discharge information following ambulatory hysterectomy.	Discharge education and provision of standardized information to support postoperative self-care.	Role description and patient-level outcome evaluation	Nurse-delivered discharge information was associated with high levels of confidence in postoperative self-care and high patient satisfaction.
Haufler K, Harrington M [21]	2011	USA	Descriptive study	To evaluate the impact of nurse-led preoperative telephone calls on reducing same-day surgical cancellations.	Preoperative telephone contact, verification of patient preparedness, and preoperative patient education.	Role description and organizational/service-level outcome evaluation	Uncontrolled before-after comparison: the authors reported a 53% reduction in the daily day-of-surgery cancellation rate, increased operating room use, and higher patient satisfaction scores.
Lo SL et al. [22]	2009	USA	Retrospective study	To evaluate the effect of diagnosis-specific discharge instructions on patient satisfaction following MAS.	Discharge education and provision of diagnosis-specific instructions for postoperative self-care.	Role description and patient-level outcome evaluation	Diagnosis-specific discharge instructions improved patient satisfaction.
Stomberg MW et al. [33]	2008	Sweden	Descriptive study	To analyze clinical practices and routines in MAS in Sweden to identify nursing interventions that could improve patient care.	Patient education, health screening, recovery assessment, and postoperative telephone follow-up.	Role description	The findings highlighted the need for greater nursing involvement to improve the quality of care.
Gilmartin J, Wright K [19]	2007	United Kingdom	Literature review *	To synthesize the evidence on ambulatory surgery and the role of nursing.	Preoperative preparation, patient education, psychological support, and discharge planning.	Contextual review evidence	Nurses play a key role in patient preparation and education, although gaps in care remain.
Digner M [34]	2007	United Kingdom	Descriptive study	To develop a telephone-based preoperative assessment system for patients undergoing MAS.	Telephone-based preoperative assessment, patient education, risk identification, and coordination of diagnostic tests and referrals.	Role description and organizational/service-level outcome evaluation	Service-development report: the author described improved theatre utilization, fewer non-attendances, and fewer hospital visits; no quantitative data or comparison group were reported.
Kingdon B, Newman K [25]	2006	USA	Descriptive study	To identify the discharge criteria used by nurses in MAS.	Clinical assessment and discharge decision-making.	Role description	Development of a discharge-criteria checklist to improve patient safety and the quality of the discharge process.
Palese A et al. [35]	2005	Italy	Quasi-experimental study	To evaluate the impact of the Nurse Case Manager model on patients undergoing MAS.	Coordination of the care pathway, patient follow-up, and serving as a contact point for postoperative concerns.	Role description and patient-level outcome evaluation	Two-ward comparison (nurse case manager vs. team nursing; *n* = 145): patients in the case-manager ward reported lower perioperative anxiety and pain and identified the unit as a point of reference for postoperative problems; pathway efficiency was described but not measured.
Guido BA [20]	2004	USA	Descriptive study	To describe the role of the Advanced Practice Nurse in an MAS unit.	Preoperative assessment, coordination of the surgical pathway, patient education, and perioperative follow-up.	Role description	Descriptive account of the advanced practice nurse role; the author stated that nurse-led preoperative care minimizes operating room delays, but no outcomes were measured.

* Note: Literature reviews [18,19] were included to map prior syntheses of the nursing role in MAS, were analyzed separately from primary empirical studies, and are not included in the frequency counts reported in Section 3.1, Section 3.1.1, Section 3.1.2 and Section 3.1.3, Table 4 and Table 5, or in the Section 4.

**Table 4:** Design, comparison, and service-level indicators of the primary studies that measured organizational or service-level outcomes (*n* = 6).

Study	Design and Comparison	Service-Level Indicator(s) Measured	Result as Reported by the Authors	Concurrent Control Group
Haufler K, Harrington M [21]	Uncontrolled before-after comparison with the previous year; one ambulatory surgery center (USA)	Daily day-of-surgery cancellation rate; operating room use	53% reduction in the daily cancellation rate; increased operating room use (not quantified); higher patient satisfaction scores	No
Wolgemuth E et al. [23]	Prospective quality-improvement project; uncontrolled before-after (6 weeks pre, 6 weeks post; 150 chart reviews)	Day-of-surgery cancellation rate	Reduction from 3.38% to 1.72%, reported as statistically significant	No
Wu M et al. [27]	Uncontrolled (self-controlled) before-after study; one day-surgery ward; 353 vs. 348 elderly patients	Waiting time for admission; waiting time for surgery; time from return to ward to discharge; compliance with audit indicators	101.8 to 89.8 h (*p* = 0.002); 15.1 to 11.1 h (*p* = 0.025); 12.3 to 11.6 h (*p* = 0.041); improved compliance with all audit indicators	No
Higueras San Román BM [30]	Descriptive longitudinal study (2018–2019); nurse-led anesthesia clinic of the MAS unit of one Spanish university hospital (Hospital Universitario Miguel Servet); 4863 patients assessed, 4013 scheduled for surgery; no intervention or comparison group	Surgical cancellation rate and causes, including nursing-related causes	Cancellation rate 2.57% (103/4013 scheduled procedures); medical causes 54.36%, patient non-attendance 19.42%, inadequate preoperative preparation (fasting or medication) 11.65%, scheduling 7.77%, other 6.80%	No
Teh AHM et al. [31]	Pre-post quality-improvement project without concurrent control; Singapore General Hospital Ambulatory Surgery Centre; 200 anesthesia records before implementation (September–December 2013) vs. 200 patients assessed by telephone after implementation (August–November 2014; ASA I–II, aged 21–50 years)	Day-of-surgery cancellation rate	Day-of-surgery cancellations decreased from 9/200 (4.5%) to 0/200 (χ^2^ test, *p* = 0.01)	No
Digner M [34]	Service-development report; one NHS trust (UK); no comparison group; no quantitative data	Theatre utilization; non-attendance (DNA); patient visits to hospital	Described as improved theatre utilization, fewer non-attendances and fewer hospital visits; not quantified	No

Palese et al. [35] compared two day-surgery wards concurrently (nurse case manager model vs. team nursing; *n* = 145) but measured only patient-level outcomes (perioperative anxiety, pain, perception of the unit as a point of reference); pathway efficiency was described qualitatively and not measured, and the study is therefore classified as “Role description and patient-level outcome evaluation” (Table 3 and Table 5).

**Table 5:** Cross-reference of included sources with the six categories of nursing roles in MAS.

Source	Evidence Level (Table 3)	C1	C2	C3	C4	C5	C6
Wolgemuth E et al. [23]	RD + OO	●			●		
Yao X et al. [26]	RD + PO		●	●			
Wu M et al. [27]	RD + OO	●	●	●	●		
Nallani R et al. [24]	RD		●		●		
Arun N et al. [29]	RD + PO	●	●			●	
Higueras San Román BM [30]	RD + OO	●	●			●	
Xu Y [28]	RD + PO	●	●	●	●	●	●
Teh AHM et al. [31]	RD + OO	●			●	●	
Lynch KM, LeFort SM [32]	RD + PO		●				
Haufler K, Harrington M [21]	RD + OO	●	●		●		
Lo SL et al. [22]	RD + PO		●				
Stomberg MW et al. [33]	RD	●	●	●			
Digner M [34]	RD + OO	●	●		●		
Kingdon B, Newman K [25]	RD		●				
Palese A et al. [35]	RD + PO			●	●	●	
Guido BA [20]	RD	●	●	●	●	●	
Primary studies, *n* (of 16)		10	13	6	9	6	1
Literature reviews (contextual evidence; not counted)							
Van Caelenberg E et al. [18]	CR		●	●	●		●
Gilmartin J, Wright K [19]	CR	●	●				

C1 = preoperative assessment and preparation; C2 = patient education and discharge planning; C3 = continuity of care and postoperative follow-up; C4 = care-process and patient-flow coordination; C5 = advanced nursing practice and nurse-led services; C6 = management, staffing, leadership, and organizational planning. RD = role description; PO = patient-level outcome evaluation; OO = organizational/service-level outcome evaluation; CR = contextual review evidence. Categories are non-mutually exclusive. Frequencies refer to the 16 primary studies; the two literature reviews are shown separately and are not counted. ● = the source was assigned to the role category (the nursing role was documented in the source); blank cell = the role was not documented.

## Data Availability

No new empirical data were generated in this study. The search documentation, the records exported from each database, and the supplementary files are openly available in the Open Science Framework repository at https://doi.org/10.17605/OSF.IO/PEH6A.

## Supplementary Materials

The following supporting information can be downloaded at: https://www.mdpi.com/article/10.3390/healthcare14193289/s1, Supplementary File S1: PRISMA-ScR checklist [16]; Supplementary Table S1: Database-specific search strategies and retrieval results; Supplementary Table S2: Co-occurrence of role categories among the primary studies.

## References

[1] Abril Á., Sevilla S., Gil M., Hernández S., Abril B., Fuertes J. (2024). Aspectos básicos de la cirugía mayor ambulatoria: Un artículo monográfico. Rev. Sanit. Investig..

[2] Nicoll J.H. (1909). The surgery of infancy. Br. Med. J..

[3] Cordero Lorenzo J.M. (2025). ASECMA 2024. Eficiencia y calidad en cirugía mayor ambulatoria. Cir. Mayor Ambulatoria.

[4] Ministerio de Sanidad y Consumo (2008). Manual de Unidad de Cirugía Mayor Ambulatoria: Estándares Y Recomendaciones.

[5] Vila-Blanco J. (2004). Desarrollo e implantación de la CMA en España: Evolución en los hospitales del INSALUD. Cir. Esp..

[6] (2026). Spanish National Health System. Key Indicators. https://inclasns.sanidad.gob.es/main.html?lang=EN.

[7] Morales García D. (2022). Cartera de Servicios en Cirugía Mayor Ambulatoria. Aumentando la Complejidad sin Poner en Riesgo la Seguridad. Cir Mayor Ambul [Internet]. https://www.asecma.org/Documentos/Articulos/0B_editorial_1.pdf.

[8] Morales-García D., García M., Lanza J., Durantez F., Grau L. (2024). DUCMA 2.0 project: Update on the current situation of the outpatient surgery units in Spain. Cir. Esp..

[9] Thoen C., Sæle M., Strandberg R., Eide P., Kinn L. (2024). Patients’ experiences of day surgery and recovery: A meta ethnography. Nurs. Open.

[10] Güven B., Sevinç C. (2024). Examining the relationship between perceived readiness for hospital discharge and quality of perioperative nursing care in ambulatory surgery patients. Med. J. Bakirkoy.

[11] Xu H., Shi Y. (2024). Effectiveness of nursing care intervention for alleviation of anxiety, pain and functional improvement amongst patients undergoing ambulatory surgery: A systematic review and meta-analysis. Pak. J. Med. Sci..

[12] Bultsma G., Schrotenboer P., Bingham C., Mussagy M. (2022). On-boarding and Orientation in an Ambulatory Surgery Center—What a Clinical Nurse Educator Can Do For You!. J. PeriAnesthesia Nurs..

[13] Morales-García D., Durantez F., Vallvey J., Grau J., Aracil X., Cimarras M., Vinagre S.G., Grau L.A.H., Fusté P., Sánchez Z.V. (2023). Training in ambulatory surgery. A pending subject in our country. Cir. Esp..

[14] Carro J., Guerrero B. (2022). Organización y funcionamiento de las unidades de Cirugía Mayor Ambulatoria. Cir. Andal..

[15] Peters M., Marnie C., Tricco A., Pollock D., Munn Z., Alexander L., McInerney P., Godfrey C.M., Khalil H. (2021). Updated methodological guidance for the conduct of scoping reviews. JBI Evid. Implement.

[16] Tricco A.C., Lillie E., Zarin W., O’Brien K.K., Colquhoun H., Levac D., Moher D., Peters M.D.J., Horsley T., Weeks L. (2018). PRISMA extension for scoping reviews (PRISMA-ScR): Checklist and explanation. Ann. Intern Med..

[17] Ouzzani M., Hammady H., Fedorowicz Z., Elmagarmid A. (2016). Rayyan—A web and mobile app for systematic reviews. Syst. Rev..

[18] Van Caelenberg E., Malfait S., De Regge M., Van Tongel A., Verhaeghe R., Joshi G., Coppens M. (2023). Staffing models A Literature Review of Staffing Models in an Ambulatory Surgery Unit Responding to Varying flow, Volume and Acuity: Identifying the Need for Further Research. Ambul. Surg..

[19] Gilmartin J., Wright K. (2007). The nurse’s role in day surgery: A literature review. Int. Nurs. Rev..

[20] Guido B. (2004). The role of a nurse practitioner in an ambulatory surgery unit. AORN J..

[21] Haufler K., Harrington M. (2011). Using nurse to patient telephone calls to reduce day-of-surgery cancellations. AORN J..

[22] Lo S., Stuenkel D., Rodriguez L. (2009). The impact of diagnosis-specific discharge instructions on patient satisfaction. J. PeriAnesth Nurs..

[23] Wolgemuth E., Glackin J., Linquist A., Dorman J., Somanath E., Canale M., Rauch E. (2025). Implementation of a Preanesthetic Telehealth Visit to Reduce Day-of-Surgery Cancellations. J. PeriAnesthesia Nurs..

[24] Nallani R., Fox C.C., Sykes K.J., Surprise J.K., Fox C.E., Reschke A.D., Simpson M.H., Polivka B.J., Villwock J.A. (2021). Pain management and education for ambulatory surgery: A qualitative study of perioperative nurses. J. Surg. Res..

[25] Kingdon B., Newman K. (2006). Determining Patient Discharge Criteria in an Outpatient Surgery Setting. AORN J..

[26] Yao X., Tai Y., Luo X., Wang K., Shang J. (2025). Postoperative feedback-oriented health education in ambulatory robot-assisted adrenalectomy: A single-center nursing intervention cohort study. J. Robot Surg..

[27] Wu M., Xia L., Zhang L., Xu Y., Cheng Y., Zhang X., Chen L. (2023). Perioperative management for elderly patients undergoing day surgery: Evidence-based practice for nursing care and day surgery. Front Med..

[28] Xu Y. (2018). Exploration of a new model of nursing management in ambulatory surgical ward. Ambulatory Surgery International Journal Covering Surgery, Anaesthesiology, Nursing and Management Issues in Day Surgery 2nd IAAS European Congress on Ambulatory Surgery.

[29] Arun N., Al-Jaham K., Alhebail S., Hassan M., Bakhit R., Paulose J., Marcus M.A.E., Ramachandran B., Lance M.D. (2021). Nurse-run preanaesthesia assessment clinics: An initiative towards improving the quality of perioperative care at the ambulatory care centre. BMJ Open Qual..

[30] Higueras San Román B. (2020). Análisis del papel de la enfermera en las suspensiones quirúrgicas del paciente intervenido en cirugía sin ingreso. Ágora Enferm..

[31] Teh A.H.M., Turner B.S., Tan S.B., Tham C.S. (2016). Effectiveness of an advanced practice nurse–led preoperative telephone assessment. J. Nurs. Care Qual..

[32] Lynch K., LeFort S. (2016). Standardized discharge information after short-stay hysterectomy and relationships with self-care confidence, perceived recovery, and satisfaction. J. Obstet. Gynecol. Neonatal Nurs..

[33] Stomberg M., Segerdahl M., Rawal N., Jakobsson J., Brattwall M. (2008). Clinical practice and routines for day surgery in Sweden: Implications for improvement in nursing interventions. J. PeriAnesth Nurs..

[34] Digner M. (2007). At your convenience: Preoperative assessment by telephone. J. Perioper. Pract..

[35] Palese A., Comuzzi C., Bresadola V. (2005). Global case management: The “nurse case manager” model applied to day surgery in Italy. Lippincott’s Case Manag..

[36] Caponnetto V., Di Nitto M., Cesare M., Iovino P., Longobucco Y., Marcomini I., Zaghini F., Alvaro R., Burgio A., Cicolini G. (2025). Factors associated with missed nursing care in home care setting: Insights from the AIDOMUS-IT multicentre study. Health Soc. Care Community.

