# Supplementary material for: Nursing Role in the Management of Major Ambulatory Surgery Units: A Scoping Review
Source: Healthcare (Basel). 2026 Oct 2;14(19):3289. doi: 10.3390/healthcare14193289 (PMC13665532; doi:10.3390/healthcare14193289)
Supplement: Supplementary file 1 [file healthcare-14-03289-s001.zip › Supplementary Table S1. Database-specific search strategies and retrieval results.pdf]

Supplementary Table S1: Database-specific search strategies and retrieval results

Database and platform	Date searched	Final search strategy	Limits applied	Records retrieved
Embase via Elsevier	31 July 2025	#1= 'major ambulatory surgery' OR 'ambulatory surgery' OR 'ambulant surgery' OR 'day surgery' OR 'outpatient surgery' OR 'same-day surgery' OR 'ambulatory surgical procedures':ab,ti<br><br>#2= 'ambulatory surgery'/exp/mj<br><br>#3= #1 OR #2<br><br>#4= management OR organization OR workflow OR efficiency OR implementation:ab,ti<br><br>#5= 'management'/exp/mj OR 'workflow'/exp/mj<br><br>#6= #4 OR #5<br><br>#7= 'nursing support' OR 'nursing interventions' OR 'nurse role' OR 'nursing care':ab,ti<br><br>#8= 'nurse'/exp/mj OR 'nursing'/exp/mj<br><br>#9= #7 OR #8<br><br>#10= #3 AND #6 AND #9<br><br>#11= #10 AND ([english]/lim OR [spanish]/lim)	English or Spanish.<br>No publication-date limit was applied during the database search.<br>The eligibility period from January 2000 to July 2025 was applied during screening.	297

MEDLINE All via Ovid	30 July 2025	1= exp *Ambulatory Surgical Procedures/<br><br>2= ('major ambulatory surgery' or 'ambulatory surgery' or 'ambulant surgery' or 'day surgery' or 'outpatient surgery' or 'same-day surgery' or 'ambulatory surgical procedures').ab,ti.<br><br>3= 1 or 2<br><br>4= exp *Case Management/ or exp *Workflow/<br><br>5= (management or organization or workflow or efficiency or implementation).ab,ti.<br><br>6= 4 or 5<br><br>7= exp *Nurses/ or exp *Nurse's Role/<br><br>8= ('nursing support' or 'nursing interventions' or 'nurse role' or 'nursing care').ab,ti.<br><br>9= 7 or 8<br><br>10= 3 and 6 and 9<br><br>11= limit 10 to (english or spanish)	English or Spanish.<br>No publication-date limit was applied during the database search.<br>The eligibility period from January 2000 to July 2025 was applied during screening.	41
CINAHL Complete via EBSCOhost	31 July 2025	S1= MM "Nurses+" OR MM "Nurses by Role+" OR MM "Nursing Care+"<br><br>S2= XB 'nursing support' OR 'nursing interventions' OR 'nurse role' OR 'nursing care'	English or Spanish.<br>No publication-date limit was applied during the database search.	336

		S3= S2 OR S1<br><br>S4= MM "Management+" OR MM "Workflow"<br><br>S5= XB management OR organization OR workflow OR efficiency OR implementation<br><br>S6= S4 OR S5<br><br>S7= XB 'major ambulatory surgery' OR 'ambulatory surgery' OR 'ambulant surgery' OR 'day surgery' OR 'outpatient surgery' OR 'same-day surgery' OR 'ambulatory surgical procedures'<br><br>S8= MM "Ambulatory Surgery"<br><br>S9= S7 OR S8<br><br>S10= S9 AND S7 AND S3<br><br>S11= S9 AND S7 AND S3, limited to English and Spanish	The eligibility period from January 2000 to July 2025 was applied during screening.	
Web of Science Core Collection, SCI-EXPANDED	31 July 2025	#1 TS=('major ambulatory surgery' OR 'ambulatory surgery' OR 'ambulant surgery' OR 'day surgery' OR 'outpatient surgery' OR 'same-day surgery' OR 'ambulatory surgical procedures')<br><br>#2 TS=(management OR organization OR workflow OR efficiency OR implementation)	English or Spanish; document type: Article; Web of Science category: Nursing.<br>No publication-date limit was applied during the database search.	248

		#3 TS=('nursing support' OR 'nursing interventions' OR 'nurse role' OR 'nursing care')<br><br>#4= #1 AND #2 AND #3<br><br>#5= #4 AND Languages=(English OR Spanish)<br><br>#6= #5 AND Document Types=(Article)<br><br>#7= #6 AND Web of Science Categories=(Nursing)	The eligibility period from January 2000 to July 2025 was applied during screening.	

Note: The final search strategies were adapted to syntax, controlled vocabulary, field codes, and search functionalities of each database platform. The publication-date eligibility criterion, defined as January 2000 to July 2025, was applied during the screening process rather than as a database search limit. The final searches yielded 922 records in total: 297 from Embase, 41 from MEDLINE, 336 from CINAHL Complete, and 248 from Web of Science Core Collection. The final Web of Science result was obtained after applying the language, document type, and Nursing category refinements. The complete search history and associated search documentation are available in the Open Science Framework repository: https://doi.org/10.17605/OSF.IO/PEH6A.
