# Supplementary material for: Nursing Role in the Management of Major Ambulatory Surgery Units: A Scoping Review
Source: Healthcare (Basel). 2026 Oct 2;14(19):3289. doi: 10.3390/healthcare14193289 (PMC13665532; doi:10.3390/healthcare14193289)
Supplement: Supplementary file 1 [file healthcare-14-03289-s001.zip › Supplementary Table S2. Co-occurrence of role categories.pdf]

Supplementary Table S2. Co-occurrence of role categories among the primary studies

Nursing Role in the Management of Major Ambulatory Surgery Units: A Scoping Review (healthcare-4596128).

Diagonal cells give the number of primary studies assigned to each category; off-diagonal cells give the number of primary studies assigned to both categories. Categories are non-mutually exclusive. The two literature reviews [18,19] are not included.

	C1	C2	C3	C4	C5	C6
C1 Preoperative assessment and preparation	10	8	4	7	5	1
C2 Patient education and discharge planning	8	13	5	6	4	1
C3 Continuity of care and postoperative follow-up	4	5	6	4	3	1
C4 Care-process and patient-flow coordination	7	6	4	9	4	1
C5 Advanced nursing practice and nurse-led services	5	4	3	4	6	1
C6 Management, staffing, leadership, and organizational planning	1	1	1	1	1	1

Number of role categories per primary study

Number of categories	Primary studies, n	References
1	3	[22], [25], [32]
2	3	[23], [24], [26]
3	7	[21], [29], [30], [31], [33], [34], [35]
4	1	[27]
5	1	[20]
6	1	[28]

Sensitivity check excluding the four studies of borderline organizational relevance [22,24,26,32] (n = 12): C1 = 10, C2 = 9, C3 = 5, C4 = 8, C5 = 6, C6 = 1.

Median number of categories per study: 3 (range 1–6). Source data: Table 5 of the main manuscript (cross-reference of included sources with the six role categories).
